# Pyroglutamate aminopeptidase 1 may be an indicator of cellular inflammatory response as revealed using a sensitive long-wavelength fluorescent probe†

**DOI:** 10.1039/c6sc00951d

**Published:** 2016-04-11

**Authors:** Qiuyu Gong, Lihong Li, Xiaofeng Wu, Huimin Ma

**Affiliations:** a Beijing National Laboratory for Molecular Sciences, Key Laboratory of Analytical Chemistry for Living Biosystems, Institute of Chemistry, Chinese Academy of Sciences Beijing 100190 China mahm@iccas.ac.cn

## Abstract

Pyroglutamate aminopeptidase 1 (PGP-1) can remove pyroglutamic acid from the N-terminus of a polypeptide, including some important anti-inflammatory proteins. Detecting the change and distribution of cellular PGP-1 in an inflammation process would be helpful to better understand the role of this enzyme. However, no report has been found on this subject, mainly due to the lack of a proper research approach. Herein, we develop such a new method by preparing a sensitive long-wavelength fluorescent probe combined with confocal fluorescence imaging. The probe, consisting of l-pyroglutamic acid and cresyl violet, exhibits high selectivity and sensitivity for PGP-1 under physiological conditions. With this probe, the up-regulation of PGP-1 in LO-2 cells under the stimulation of Freund's incomplete adjuvant and lipopolysaccharide (two main immunopotentiators) is revealed for the first time, and this up-regulation is also observed in typical phagocytic RAW264.7 cells, as evidenced by western blot and inhibition assays. Studies on the distribution of PGP-1 in cells using our probe showed that most PGP-1 is located in the cytoplasm, which is further supported by an immunofluorescence assay. Moreover, the inflammatory response induced by the immunopotentiators in either RAW264.7 or LO-2 cells is confirmed by measuring tumor necrosis factor alpha (a common inflammatory factor). The above findings indicate that cellular inflammation is accompanied by an increase in PGP-1, and PGP-1 may serve as a new indicator of cellular inflammatory response.

## Abstract 2

Pyroglutamate aminopeptidase 1 may be an indicator of cellular inflammatory response as revealed using a sensitive long-wavelength fluorescent probe.

## Graphical abstract

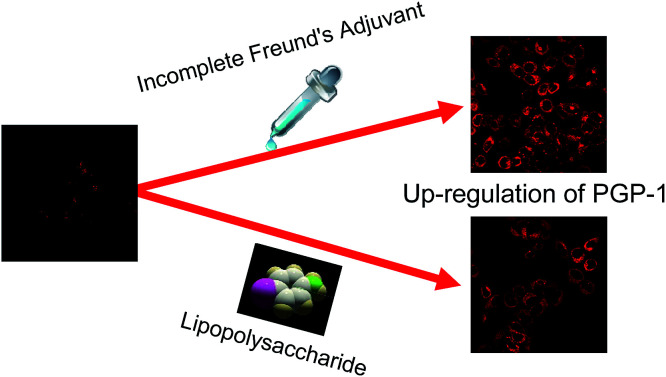

## Introduction

**Fig. 1:**
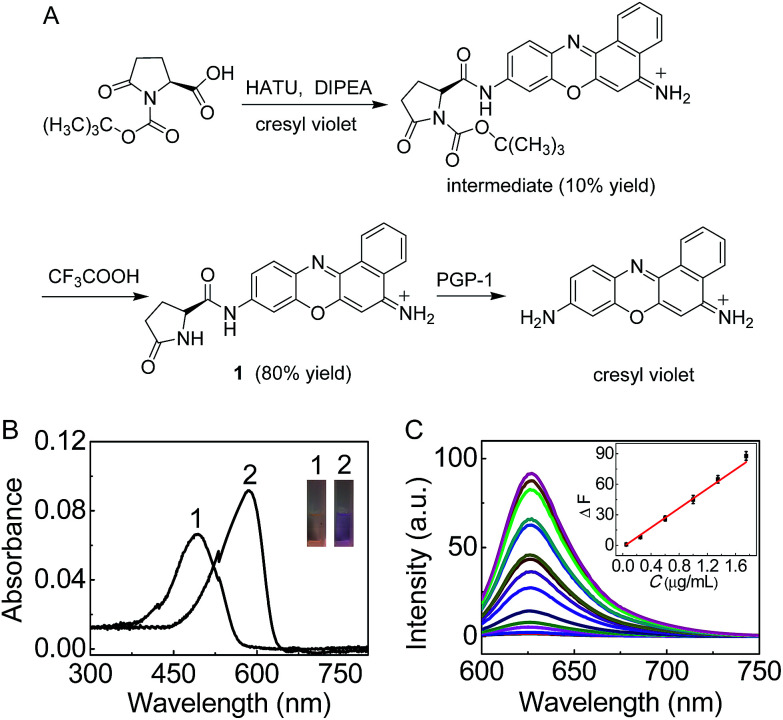
(A) Synthesis of probe 1 and its reaction with PGP-1. (B) Absorption spectra of 1 (5 μM) before (curve 1) and after (curve 2) the reaction with PGP-1 (2 μg mL^−1^) at 37 °C in 6.7 mM PBS (pH 7.4). The inset shows the corresponding color change. (C) Fluorescence response of 1 (5 μM) to PGP-1 at different concentrations (from bottom to top: 0–5 μg mL^−1^). The inset shows the plot of Δ*F versus* the PGP-1 concentration. *λ*_ex/em_ = 585/625 nm.

Pyroglutamate aminopeptidase 1 (PGP-1, EC 3.4.19.3) is a type of enzyme that cleaves the peptide bond of pyroglutamic acid linked to the N-terminal end of a protein, including some important anti-inflammatory proteins like immunoglobulin.^1–5^ On the other hand, inflammation, a protective response that involves immune cells, blood vessels and molecular mediators, plays a significant role in many diseases,^6^ on which considerable effort has already been expended.^7–9^ However, it is unknown whether PGP-1 has any relationship with the inflammatory process in cells. Without doubt, detecting the change and distribution of cellular PGP-1 would be helpful to understand this issue. To the best of our knowledge, nevertheless, no report has been found on this subject, mainly due to the lack of a proper research approach. Herein, we develop such a new method by preparing a sensitive long-wavelength fluorescent probe combined with confocal fluorescence imaging. The probe (1; Fig. 1(A)), consisting of cresyl violet and l-pyroglutamic acid (as a recognition moiety), exhibits not only long analytical wavelengths (*λ*_ex/em_ = 585/625 nm) but also high selectivity and sensitivity for PGP-1 under physiological conditions. Using this probe, the up-regulation of PGP-1 in LO-2 cells under the treatment of immunopotentiators is revealed for the first time, and this up-regulation is also found in typical phagocytic RAW264.7 cells, as confirmed using western blot and inhibitor experiments. Interestingly, studies on the distribution of PGP-1 in cells using our probe reveal that most PGP-1 may locate in the cytoplasm, and this is further verified using an immunofluorescence assay. In addition, the inflammatory response induced by the immunopotentiators in either RAW264.7 or LO-2 cells is validated by measuring the change in tumor necrosis factor alpha (TNF-α is a common inflammatory factor). Below we report these results.

## Results and discussion

### Spectroscopic properties and analytical performance of probe 1

On the basis of our previous experience,^10,11^ we first prepared 1 (Fig. 1(A)) as a sensitive long-wavelength fluorescent probe for PGP-1. Cresyl violet with long-wavelength features was chosen as a fluorescent skeleton, because the substitution of its amino group usually leads to complete fluorescence quenching,^11*b*^ which would favor a low background signal and thus sensitive detection. As shown in Fig. 1(A), probe 1 can be readily prepared by treating cresyl violet with protected l-pyroglutamic acid, and then removing the protecting group in the presence of trifluoroacetic acid (ESI†). The obtained probe was well characterized using NMR and mass spectral analysis (Fig. S1–S4, ESI†).

The spectroscopic properties and analytical performance of probe 1 were then studied in detail. As shown in Fig. 1, probe 1 itself, with a maximum absorption at 490 nm, exhibits extremely low background fluorescence (quantum yield, <0.01). However, under the optimized conditions (reaction for 30 min at 37 °C and pH 7.4; Fig. S5 and S6 in the ESI†) the probe produces a good linear fluorescence off–on response to PGP-1 in the concentration range of 0.05–1.75 μg mL^−1^ (inset of Fig. 1(C)), with an equation of Δ*F* = 47.6 × *C* (μg mL^−1^) − 1.65 (*R* = 0.995), where Δ*F* is the difference in the fluorescence intensity of 1 after and before reacting with PGP-1. The detection limit^12^ is determined to be 5.6 ng mL^−1^ of PGP-1, which is more sensitive than that (14.6 ng mL^−1^; Fig. S7, ESI†) of a commercial probe (note that the commercial probe is unsuitable for cell imaging due to its rather short wavelengths, *λ*_ex/em_ = 340/440 nm). Notably, the probe also exhibits rather high selectivity and good biocompatibility (Fig. S8 and S9†). It is worth noting that the absorption and fluorescence spectra from the reaction system accord well with those from cresyl violet.^11^ This suggests that the enzymatic cleavage reaction of 1 by PGP-1 occurred and caused the release of cresyl violet (Fig. 1(A)) with a fluorescence quantum yield of 0.51, which was further verified using mass spectral analysis (*m*/*z* = 262.0973 [M]^+^; Fig. S10†). According to the Michaelis–Menten equation (Fig. S11†),^12,13^ the corresponding Michaelis constant (*K*_m_) and the maximum of the initial reaction rate (*V*_max_) for the present enzymatic reaction were determined to be 120 μM and 0.25 μM s^−1^. Moreover, inhibitor experiments with iodoacetamide (Fig. S12†) also supported that the fluorescence off–on response of the probe arises from the enzymatic action of PGP-1.

### Detection of the up-regulation of PGP-1 in living cells under the stimulation of immunopotentiators

**Fig. 2:**
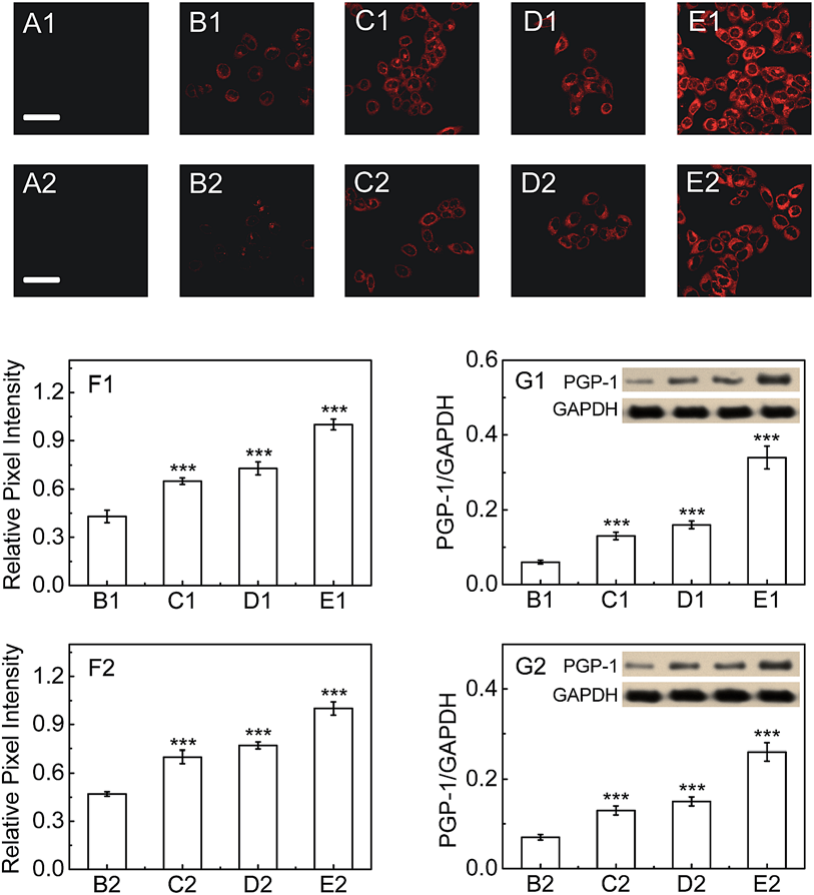
Fluorescence images of PGP-1 in LO-2 cells. (A1 and A2) LO-2 cells only. (B1–E1) The cells were pretreated with FIA at varied concentrations (0, 0.05, 0.1 and 0.25%) at 37 °C for 24 h, and then incubated with the probe (5 μM) for 30 min. (B2–E2) The cells were pretreated with LPS at varied concentrations (0, 0.2, 0.5 and 1.0 μg mL^−1^) at 37 °C for 16 h, and then incubated with the probe (5 μM) for 30 min. Scale bar, 20 μm. (F1 and F2) Relative pixel intensity measurements (*n* = 3) from the above corresponding images using the software ImageJ. The pixel intensities from images E1 and E2 are defined as 1.0 (***p < 0.001, as compared with the control, two-sided Student's *t*-test). (G1 and G2) The changes in PGP-1 for the above corresponding cells determined using western blot analysis (the molecular weight of PGP-1 is determined to be 23 kDa; glyceraldehyde-3-phosphate dehydrogenase, GAPDH, as a protein standard).

Next, we explore whether PGP-1 has a relationship with an inflammatory process in cells by detecting the change and distribution of cellular PGP-1 using probe 1 combined with confocal fluorescence imaging. As shown in Fig. 2, the fluorescence intensity of LO-2 cells treated with immunopotentiators [Freund's incomplete adjuvant (FIA), or lipopolysaccharide (LPS)]^14^ gradually increases with increasing the immunopotentiator concentration in the range of 0–0.25% (v/v) FIA (images B1–E1, and Fig. 2F1) and 0–1 μg mL^−1^ LPS (images B2–E2, and Fig. 2F2). This fluorescence increase reflects the up-regulation of the intracellular PGP-1 concentration, which is clearly confirmed using western blot analysis (see Fig. 2G1 and G2). The studies on the effect of the incubation time of FIA or LPS revealed that the intracellular fluorescence intensity, representing the PGP-1 concentration, rises in a time-dependent manner (see images B1–E1 and panel G1 in Fig. S13 in the ESI† for FIA; images B2–E2 and panel G2 in Fig. S13† for LPS). Moreover, this rise in intracellular PGP-1 is further supported by both the inhibitor experiments (images F1 and F2 in Fig. S13 and S14†) and western blot assay (Fig. S14†). It should be pointed out that the fluorescence of the reaction system of probe 1 with PGP-1 is scarcely affected by FIA or LPS itself (Fig. S15†). In addition, under the same conditions we also observed the up-regulation of PGP-1 in HepG2 cells (Fig. S16†), possibly suggesting a universal phenomenon of a PGP-1 increase in inflammatory cells.

**Fig. 3:**
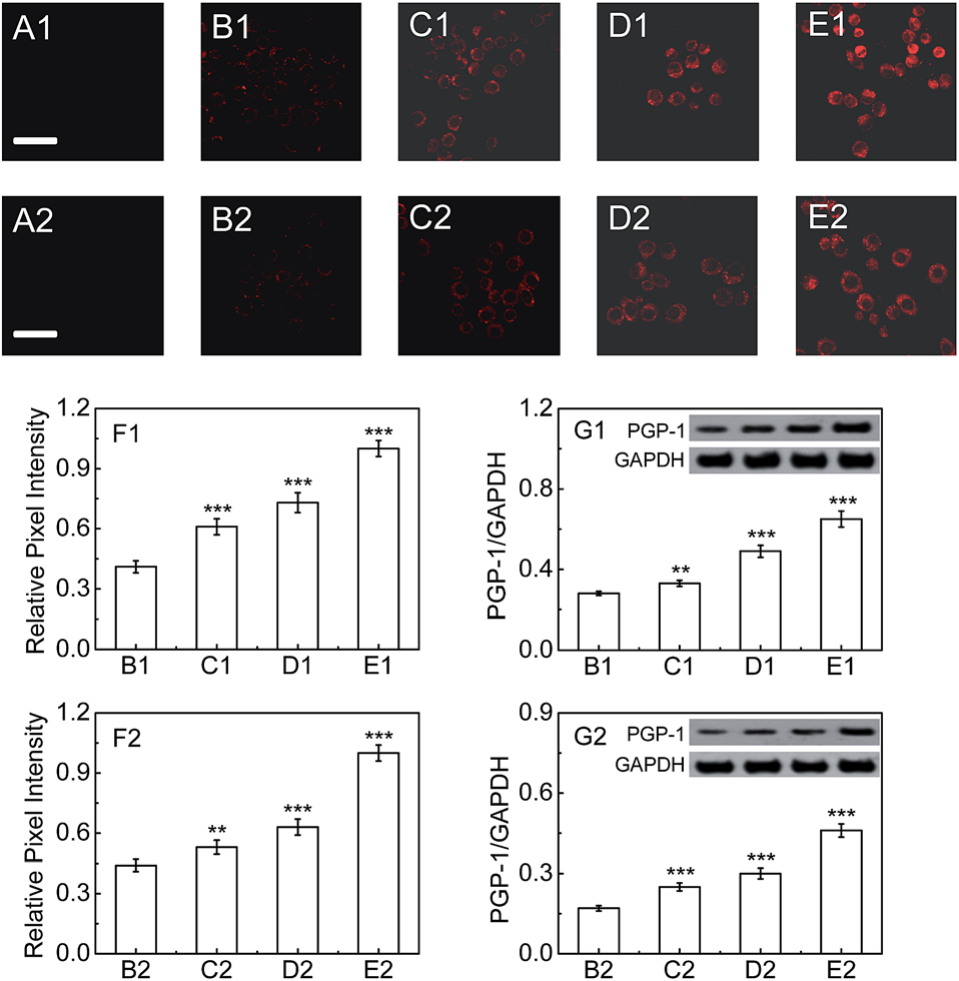
Fluorescence images of PGP-1 in RAW264.7 cells. (A1 and A2) RAW264.7 cells only. (B1–E1) The cells were pretreated with FIA at varied concentrations (0, 0.05, 0.1 and 0.25%) at 37 °C for 24 h, and then incubated with the probe (5 μM) for 30 min. (B2–E2) The cells were pretreated with LPS at varied concentrations (0, 0.2, 0.5 and 1.0) for 16 h, and then incubated with the probe (5 μM) for 30 min. Scale bar, 20 μm. (F1 and F2) Relative pixel intensity measurements (*n* = 3) from the above corresponding images. The pixel intensities from images E1 and E2 are defined as 1.0 (**p < 0.01, ***p < 0.001, as compared with the control, two-sided Student's *t*-test). (G1 and G2) The changes in PGP-1 for the above corresponding cells determined using western blot analysis.

In order to further verify this phenomenon, RAW264.7 cells, a typical phagocytic cell line,^15^ were also treated under the same conditions. As can be seen from Fig. 3, the fluorescence intensity of RAW264.7 cells treated with FIA or LPS gradually increases with increasing the immunopotentiator concentration (images B1–E1, and Fig. 3F1 for FIA; images B2–E2, and Fig. 3F2 for LPS). The fluorescence increase indicates the rise of the intracellular PGP-1 concentration, which is again proved using western blot analysis (Fig. 3G1 and G2). Therefore, it is suggested that cells, whether normal, phagocytic, or non-phagocytic,^15*b*,16^ exhibit the PGP-1 up-regulation in an inflammation process, indicating the universality of this behavior.

### Location of PGP-1

**Fig. 4:**
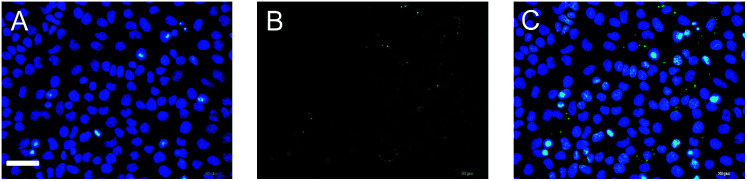
Immunofluorescence images of LO-2 cells. (A) The cells were stained with 4′,6-diamidino-2-phenylindole (a nucleus-specific dye). The blue fluorescence area represents the cell nucleus. (B) The cells were stained with fluorescein isothiocyanate that was linked to the antibody of PGP-1. The green fluorescence area, mainly appearing in the cytoplasm, represents PGP-1 in the cells. (C) The overlapped image of images A and B. Scale bar 20 μm.

Interestingly, we found that the fluorescence mainly appears in the cytoplasm of the inflammatory cells, which may result from the dominant distribution of PGP-1 in the cytoplasm. To ascertain this, we performed an immunofluorescence assay (a technique that utilizes fluorescent-labeled antibodies to detect specific target antigens).^17^ As shown in image (C) in Fig. 4, the blue area (image A), representing the cell nucleus, scarcely overlaps with the green area (image B) that reflects the location of PGP-1, which suggests that most PGP-1 indeed distributes in the cytoplasm. All the above results indicate that FIA and LPS can induce the up-regulation of intracellular PGP-1 and our probe can be used to visualize not only such an increase in PGP-1 but also the distribution of PGP-1 in cells, which however cannot be realized using western blot assay.

### Measuring the inflammatory response

**Fig. 5:**
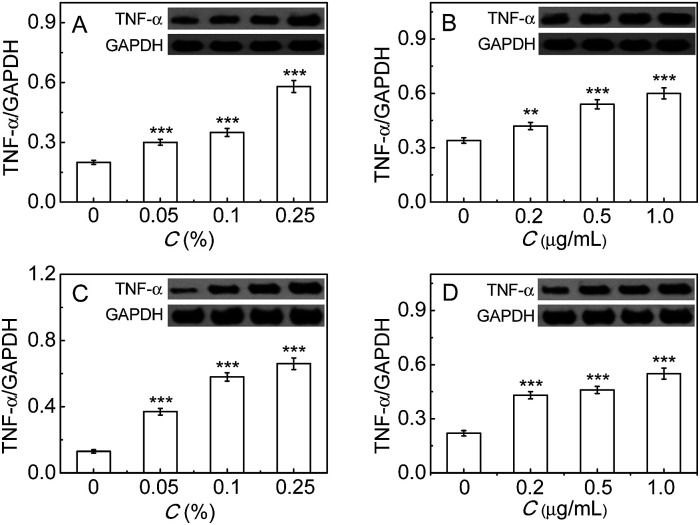
Western blot analysis of TNF-α. (A and B) The change of TNF-α in LO-2 cells under treatment with FIA (0–0.25%) and LPS (0–1.0 μg mL^−1^), respectively. (C and D) The change of TNF-α in RAW264.7 cells under treatment with FIA (0–0.25%) and LPS (0–1.0 μg mL^−1^), respectively. The molecular weight of TNF-α was determined to be 26 kDa (**p < 0.01, ***p < 0.001, as compared with the control, two-sided Student's *t*-test).

Finally, the inflammatory response induced by the immunopotentiators in either RAW264.7 or LO-2 cells was studied by measuring the common inflammatory factor TNF-α, whose up-regulation reflects the cellular inflammatory response enhancement.^9*c*,18^ As can be seen from Fig. 5, TNF-α increases with the increase of FIA or LPS, implying the enhancement of the inflammatory response. All the above findings indicate that PGP-1 may serve as an indicator of cellular inflammatory response. The exact mechanism underlying the up-regulation of intracellular PGP-1 needs to be investigated in future research, but a possible reason may be due to the fact that PGP-1 can cleave the N-terminal pyroglutamic acid in some important anti-inflammatory proteins like immunoglobulin.

## Conclusions

In conclusion, we have developed a new fluorescent PGP-1 probe, which exhibits good selectivity and high sensitivity. Using the probe combined with confocal fluorescence imaging, the up-regulation of intracellular PGP-1 in inflammatory LO-2 and RAW264.7 cells has been revealed, which is further confirmed using western blot analysis. The distribution of PGP-1 in the cytoplasm is also successfully imaged. Importantly, by measuring the inflammatory factor TNF-α, we propose for the first time that PGP-1 may serve as a new indicator of cellular inflammatory response. Besides, it is believed that with our probe more biological functions of PGP-1 may be revealed.

## Supplementary Material

SC-007-C6SC00951D-s001

## References

[cit1] Chatterton D. E. W., Nguyen D. N., Bering S. B., Sangild P. T. (2013). Int. J. Biochem. Cell Biol..

[cit2] Suzuki Y., Motoi H., Sato K. (1999). J. Agric. Food Chem..

[cit3] Awadé A. C., Cleuziat P., Gonzalees T., Robert-Baudouy J. (1994). Proteins: Struct., Funct., Genet..

[cit4] Meqias M. J., Alba-Araquez F., Luna J. D., Vives F., Ramirez-Sanchez M. (2015). Endocr. Regul..

[cit5] Abe K., Fukuda K., Tokui T. (2004). Biol. Pharm. Bull..

[cit6] Chun M. F., Chia W. T., Wan W. L., Lin Y. J., Sung H. W. (2015). J. Am. Chem. Soc..

[cit7] Skwarczynski M., Toth I. (2016). Chem. Sci..

[cit8] Esposito E., Cuzzocrea S. (2009). Curr. Med. Chem..

[cit9] Bai A. P., Moss A., Rothweiler S., Longhi M. S., Wu Y., Junger W. G., Robson S. C. (2015). Nat. Commun..

[cit10] Li X. H., Gao X. H., Shi W., Ma H. M. (2014). Chem. Rev..

[cit11] Wan Q. Q., Song Y. C., Li Z., Gao X. H., Ma H. M. (2013). Chem. Commun..

[cit12] Li L. H., Shi W., Wang Z., Gong Q. Y., Ma H. M. (2015). Anal. Chem..

[cit13] Ge G. B., Ning J., Hu L. H., Dai Z. R., Hou J., Cao Y. F., Yu Z. W., Ai C. Z., Gu J. K., Ma X. C., Yang L. (2013). Chem. Commun..

[cit14] Ostberg K. L., Russell M. W., Murphy T. F. (2008). Mucosal Immunol..

[cit15] Kong L., Ge B. X. (2008). Cell Res..

[cit16] Dai B. H., Geng L., Wang Y., Sui C. J., Xie F., Shen R. X., Shen W. F., Yang J. M. (2013). Cell Death Dis..

[cit17] Luchsinger L. L., Almeida M. J., Corrigan D. J., Mumau M., Snoeck H. W. (2016). Nature.

[cit18] Miller A. H., Maletic V., Raison C. L. (2009). Biol. Psychiatry.

