# Supplementary material for: Pyroglutamate aminopeptidase 1 may be an indicator of cellular inflammatory response as revealed using a sensitive long-wavelength fluorescent probe
Source: Chem Sci. 2016 Apr 11;7(7):4694–7. doi: 10.1039/c6sc00951d (PMC6013796; doi:10.1039/c6sc00951d)
Supplement: SC-007-C6SC00951D-s001 [file SC-007-C6SC00951D-s001.pdf]

Electronic Supplementary Material (ESI) for Chemical Science.
This journal is © The Royal Society of Chemistry 2016

Electronic Supplementary Information for:

Pyroglutamate Aminopeptidase 1 May be an Indicator of Cellular Inflammatory Response as Revealed by a Sensitive Long-Wavelength Fluorescent Probe

Qiuyu Gong, Lihong Li, Xiaofeng Wu and Huimin Ma*

Beijing National Laboratory for Molecular Sciences, Key Laboratory of Analytical Chemistry for Living Biosystems, Institute of Chemistry, Chinese Academy of Sciences, Beijing 100190, China. E-mail: mahm@iccas.ac.cn

1. Experimental

Reagents. N-(tert-Butoxycarbonyl)-L-pyroglutamic acid was obtained from Tokyo Chemical Industry CO., LTD. N,N-Diisopropylethylamine (DIPEA) and cresyl violet acetate were purchased from Acros Organics. O-(7-Aza-1H-benzotriazol-1-yl)-N,N,N',N'-tetramethyluronium hexafluorophosphate (HATU) and trifluoroacetic acid (HPLC grade) were purchased from Alfa Aesar Chemicals. Freund's incomplete adjuvant (FIA), lipopolysaccharides (LPS), dimethyl sulfoxide (DMSO), 3-(4,5-dimethylthiazol-2-yl)-2,5-diphenyltetrazolium bromide (MTT), iodoacetamide, reduced glutathione (GSH), leucine aminopeptidase (LAP), esterase, V8 protease, prolidase, trypsin, thrombin, dipeptidyl peptidase IV (DPPIV) and commercial PGP-1 probe (L-pyroglutamic acid 7-amido-4-methylcoumarin) were purchased from Sigma-Aldrich. Pyroglutamate aminopeptidase 1 (PGP-1; molecular weight, 23 kDa) was obtained from State Key Laboratory of Antibody Medicine and Targeted Therapy, Shanghai, China. Roswell Park Memorial Institute-1640 medium (RPMI-1640), Dulbecco's modified Eagle's medium (DMEM), calf serum, LO-2, HepG2 and RAW264.7 cell lines were purchased from KeyGEN BioTECH Co. LTD, Nanjing, China. A 6.7 mM phosphate buffered saline (PBS: 9 g/L NaCl, 795 mg/L Na_2HPO_4 and 144 mg/L KH_2PO_4) of pH 7.4 was obtained from Invitrogen. Fetal bovine serum was purchased from Invitrogen Corporation. Proteins were pure as judged by Coomassie-stained SDS-polyacrylamide gel electrophoresis (SDS-PAGE). RIPA (radio immunoprecipitation assay) lysis buffer (CW2333) was purchased from CWbiotech. Co. Ltd, Beijing, China. Agarose, poly(vinylidene fluoride) membranes (PVDF membranes), anti-TNF-α antibody, gel electrophoresis kits, western blot kits, enhanced chemiluminescence kits (ECL) and Braford protein assay kits were purchased from KeyGEN BioTECH Co. Ltd, Nanjing, China. Anti-PGP-1 antibody and pyroglutamate aminopeptidase 2 (PGP-2) were purchased from Proteintech^{TM}, USA. All other chemicals used were local products of analytical grade. Ultrapure water (over 18 MΩ·cm) from a Milli-Q reference system (Millipore) was used throughout. The stock solution (1.0 mM) of probe **1** was prepared by dissolving requisite

amount of it in DMSO. Stock solutions of other substances were prepared by dissolving in PBS or water.

Apparatus. Fluorescence measurements were made on a Hitachi F-4600 fluorimeter (Tokyo, Japan). UV-vis absorption spectra were measured in 1×1 cm quartz cells with a TU-1900 spectrophotometer (Beijing, China). A model HI-98128 pH-meter (Hanna Instruments Inc., USA) was used for pH measurements. ^{1}H NMR and ^{13}C NMR spectra were measured with a Bruker Avance 400 or 600 spectrometer in CD_3OD or DMSO-d_6. Electrospray ionization mass spectra (ESI-MS) were measured with an LC-MS 2010A (Shimadzu) instrument. High resolution electrospray ionization mass spectra (HR-ESI-MS) were recorded on an APEX IV FTMS instrument (Bruker, Daltonics). Cell imaging experiments were operated on a FV 1000-IX81 confocal laser scanning microscope (Olympus, Japan). The fluorescence quantum yield (Φ) was determined by using fluorescein (Φ = 0.85 in 0.1 M NaOH) as a standard (Zhang et al, *J. Am. Chem. Soc.* 2011, **133**, 14109). The western blot signal was detected using an ECL kit. Image processing was made with Olympus ImageJ software (National Institutes of Health, USA). Cytotoxicity assay was made on microtiter plate assay system (Molecular Devices, USA).

Synthesis of probe 1. N-(tert-Butoxycarbonyl)-L-pyroglutamic acid (229 mg, 1.0 mmol), HATU (570 mg, 1.5 mmol) and DIPEA (2.0 mmol, 435 μL) were added to 30 mL of acetonitrile at 0 °C. After stirring for 40 min, cresyl violet acetate (321 mg 1.0 mmol) was added slowly, and the reaction mixture was further stirred at room temperature for 3 h. Then, the mixture was diluted with ethyl acetate, and washed three times with water (100 mL×3). The solvent was removed by evaporation under reduced pressure, and the crude product was purified by silica gel chromatography eluted with CH_2Cl_2/methanol (v/v, 20/1), affording the intermediate as a dark red solid (47.3 mg, yield 10%). The ^{1}H NMR and ^{13}C NMR spectra of the intermediate are shown below in Fig. S1 and Fig. S2, respectively. ^{1}H NMR (400 MHz, CD_3OD): δ 8.77-8.79 (d, 1H), 8.33-8.35 (d, 1H), 8.03 (s, 1H), 7.82-7.88, (m, 3H), 7.49-7.51 (d, 1H), 6.68 (s, 1H), 3.67 (s, 1H), 2.44-2.75 (m, 3H), 2.05-2.23 (m, 2H), 1.47 (s, 9H). ^{13}C NMR (100 MHz, CD_3OD): δ 176.6, 172.4, 150.7, 133.4, 132.6, 130.5, 126.2, 125.2, 84.6, 71.0, 61.9, 36.3, 32.8, 31.9, 30.5, 30.3, 30.1, 30.0, 27.9, 26.6, 23.4, 22.6, 14.1. HR-ESI-MS, calcd for $C_{26}H_{25}N_4O_5$ $[M]^+$: *m/z* 473.1819; found: *m/z* 473.1817.

Then, probe **1** was prepared as follows. Trifluoroacetic acid (2.5 mL) in CH_2Cl_2 (2.5 mL) was added dropwise to a solution of the above intermediate in 5 mL of CH_2Cl_2 at 0 °C, and the reaction mixture was stirred at room temperature for 3 h. The solvent was removed by evaporation under reduced pressure, and the crude product was purified by flash silica gel chromatography eluted with CH_2Cl_2/methanol (v/v, 10/1), affording probe **1** as a crimson solid (29 mg, yield 80%). The ^{1}H NMR and ^{13}C NMR spectra of probe **1** are shown below in

Fig. S3 and Fig. S4, respectively. ^{1}H NMR (400 MHz, DMSO-d_6): δ 10.87 (s, 1H), 8.45-8.55 (d, 2H), 7.98 (s, 1H), 7.79 (s, 1H), 7.53-7.67 (d, 3H), 6.60 (s, 1H), 3.53 (s, 1H), 1.99-2.24 (m, 5H). ^{13}C NMR (150 MHz, DMSO-d_6): δ 177.9, 172.7, 158.5, 158.3, 145.0, 142.3, 131.9, 131.0, 125.2, 124.9, 118.9, 116.9, 105.8, 72.8, 60.8, 56.9, 29.7, 29.5, 25.8, 21.7, 14.5. HR-ESI-MS, calcd for $C_{21}H_{17}N_4O_3$ $[M]^+$: *m/z* 373.1295; found: *m/z* 373.1295.

General procedure for PGP-1 detection. Unless otherwise stated, all the fluorescence measurements were made according to the following procedure. In a test tube, 5 μL of stock solution of probe **1** and appropriate volume of PBS were mixed, followed by addition of an appropriate volume of PGP-1 or other substance solutions. The mixed solution was adjusted to 1 mL with PBS. After incubation at 37 °C for 30 min, the reaction solution was transferred to a quartz cell of 1-cm optical length to measure fluorescence with $\lambda_{ex/em}$ = 585/625 nm (both excitation and emission slit widths were set to 5 nm). For absorbance measurements, 2 mL of reaction solution was prepared and used. At the same time, a blank solution without PGP-1 was prepared and measured under the same conditions for comparison. Data are expressed as mean ± standard deviation (SD) of three separate measurements.

Cell imaging. Cells (LO-2, HepG2 or RAW264.7) were treated at 37 °C for different periods of time (0-24 h or 0-16 h) with various concentrations of FIA (0-0.25%, v/v) or LPS (0-1.0 μg/mL). For inhibitor experiments, cells were treated according to the above method and then incubated with iodoacetamide (5 nM and 50 nM) in Petri dishes. Before cell imaging, the culture media were removed, and the cells were washed using DMEM or RPMI-1640 for three times. Then, the cells were incubated with the probe (5 μM) at 37 °C for 30 min in DMEM or RPMI-1640, washed with DMEM or RPMI-1640 to remove the free probe, and finally subjected to fluorescence imaging. Unless otherwise noted, data are expressed as mean ± standard deviation (SD) of three separate measurements.

Cytotoxicity assay. The cytotoxicity of probe **1** was tested on LO-2 cells using a standard MTT assay, as described previously (Gong et al, *Chem. Sci.* 2016, **7**, 788).

Preparation of cell lysates. The cell lysates were prepared according to our previous method (Gong et al, *Chem. Sci.* 2016, **7**, 788).

Western blot. Unless otherwise stated, all the western blot analyses were made by using GAPDH (glyceraldehyde-3-phosphate dehydrogenase) as a protein standard. In brief, cells (LO-2 or RAW264.7, including control group, and FIA, LPS and inhibitor treated groups) were lysed with RIPA buffer, and the cell lysates were diluted 10 times with PBS to obtain a solution of about 0.5 mg/mL of total proteins. The diluted cell lysates were incubated with Anti-PGP-1 or Anti-TNF-α antibody for 3 h at room temperature, and then incubated with Protein A/G Agarose for 1.5 h at room temperature. The agarose beads were pelleted by

centrifugation at 4 °C (14000 rpm for 5 min), and the PGP-1 or TNF-α-depleted supernatant was collected and preserved at -70 °C. For western blot analysis, equal amounts of protein in cell lysates were separated by SDS-PAGE and electroblotted onto PVDF membranes. After blocking with 5% skim milk in TBST (20 mM Tris, pH 7.6, 140 mM NaCl, and 0.1% Tween-20) at room temperature for 1.5 h, the membranes were incubated with the corresponding primary antibodies at 4 °C overnight. The membranes were washed with TBST for three times and further incubated with the horseradish peroxidase-conjugated secondary antibodies at room temperature for 2 h. Finally, the signal was detected using an ECL kit. The grayscale value was analyzed by Gel-Pro32 software.

Immunofluorescence assay. The immunofluorescence images were recorded according to the previous methods (Zhu et al, *Nat. Commun.* 2016, **7**, 10080; Ensink et al, *Anal. Chem.* 2015, **87**, 9715) in KeyGEN BioTECH Co. Ltd, Nanjing, China.

Statistical tests. The *t* analysis was operated according to our previous method (Gong et al, *Chem. Sci.* 2016, **7**, 788).

2. Supporting Figures

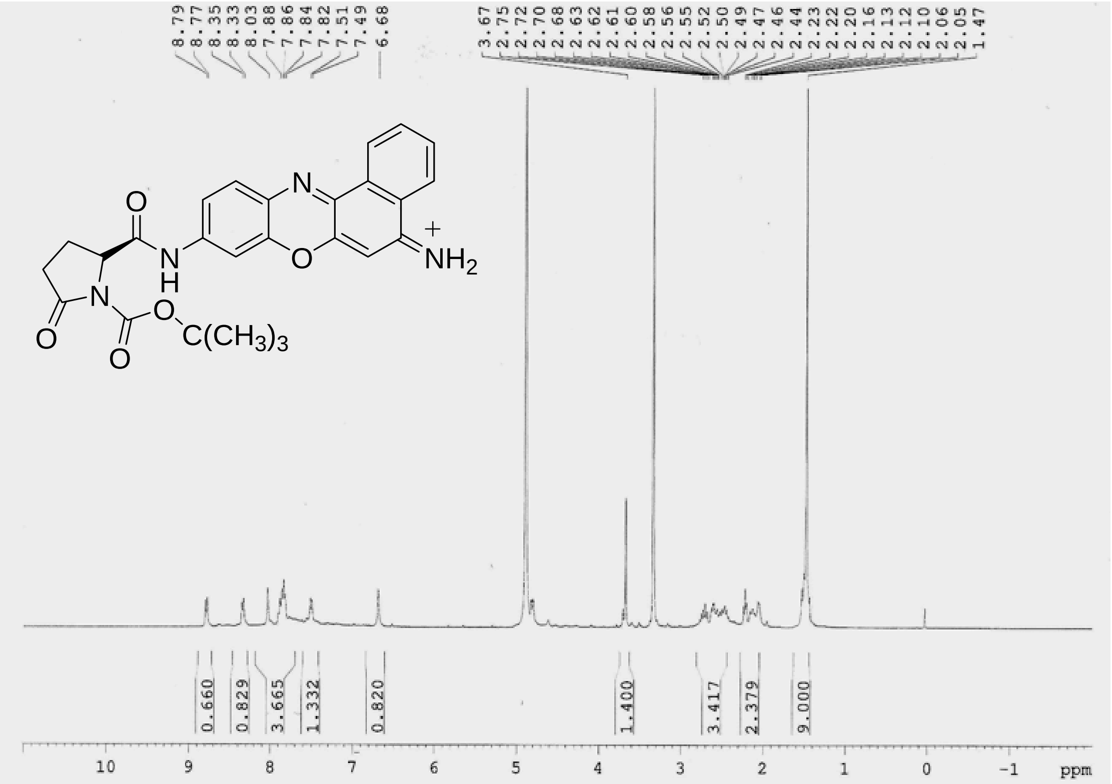

Fig. S1 ^{1}H NMR spectrum of intermediate (400 MHz, CD_3OD, 298 K).

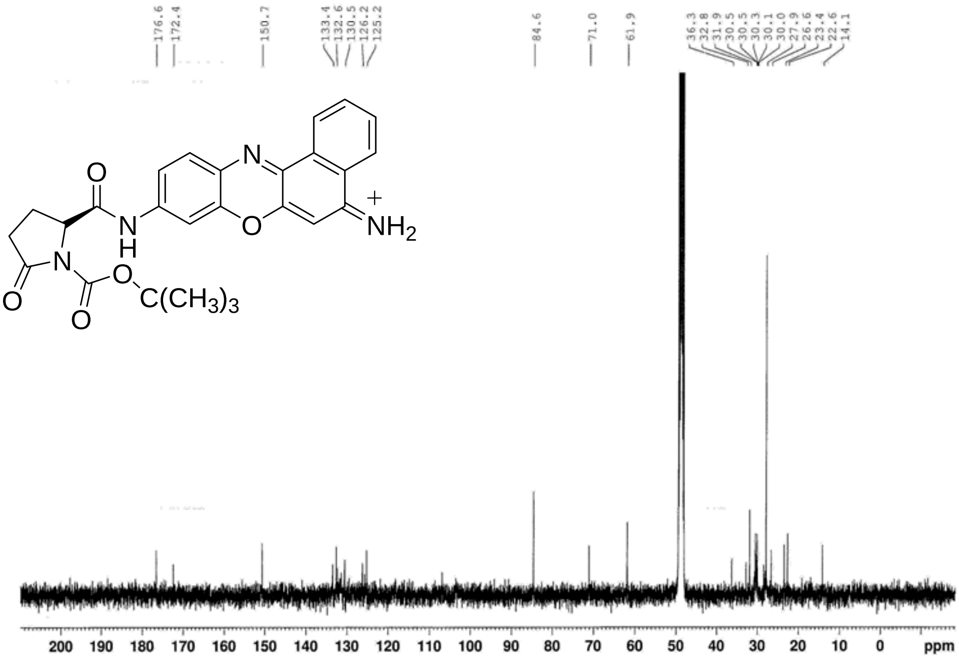

Fig. S2 ^{13}C NMR spectrum of intermediate (100 MHz, CD_3OD, 298 K).

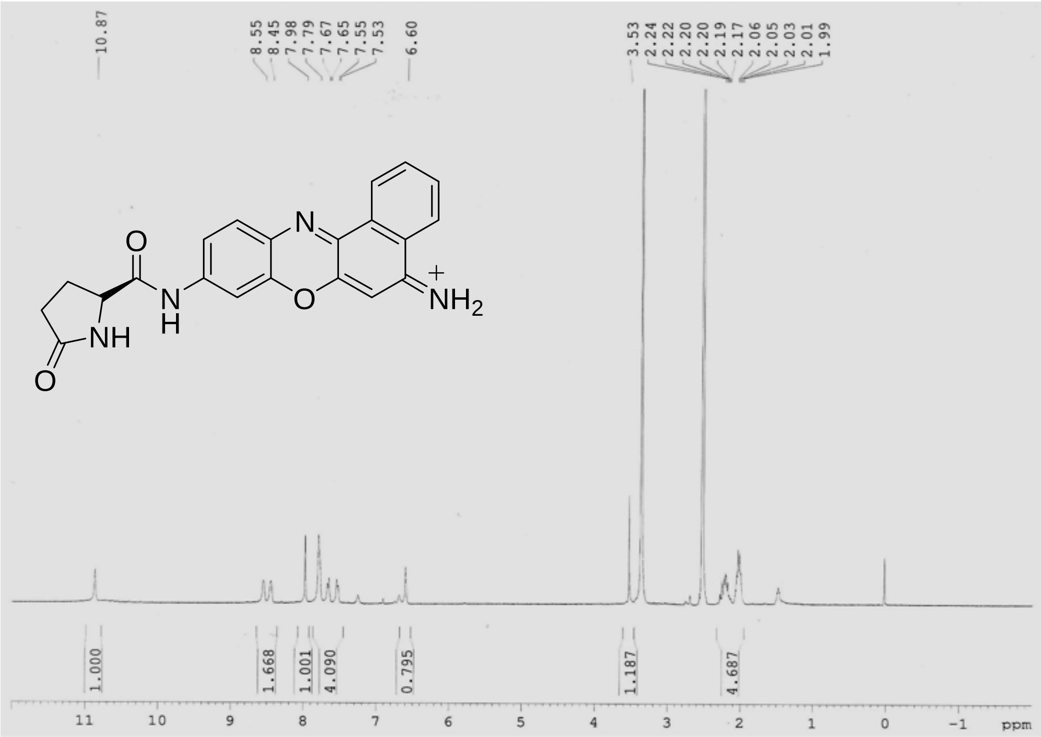

Fig. S3 ^{1}H NMR spectrum of probe **1** (400 MHz, DMSO-d_6, 298 K).

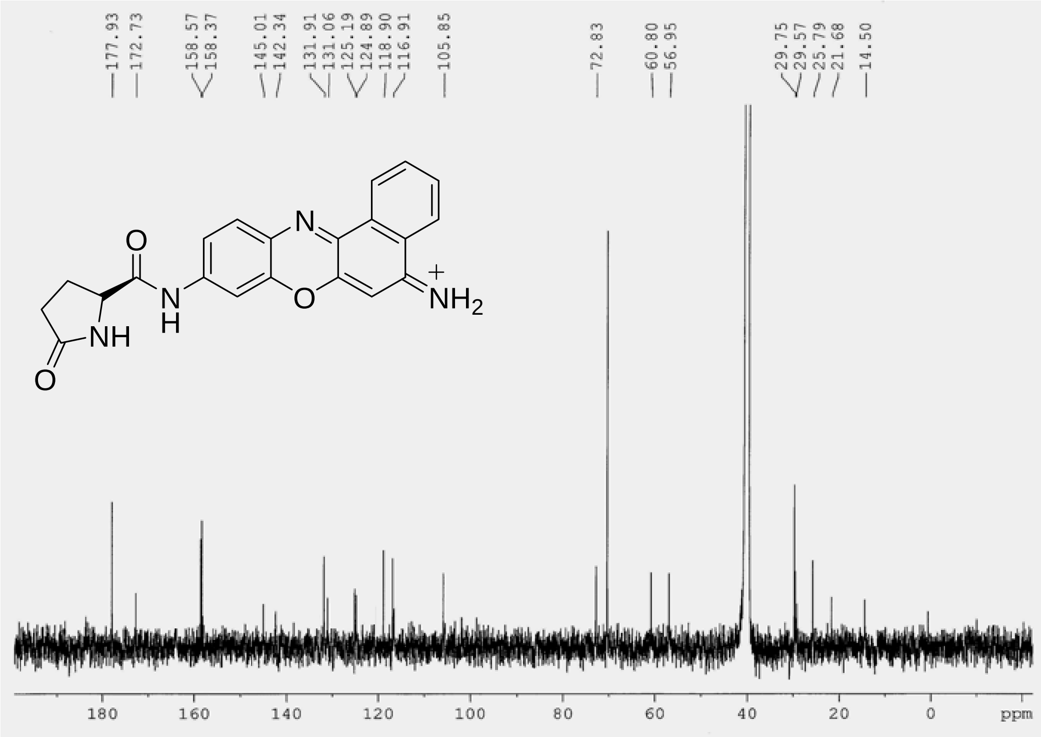

Fig. S4 ^{13}C NMR spectrum of probe **1** (150 MHz, DMSO-d$_6$, 298 K).

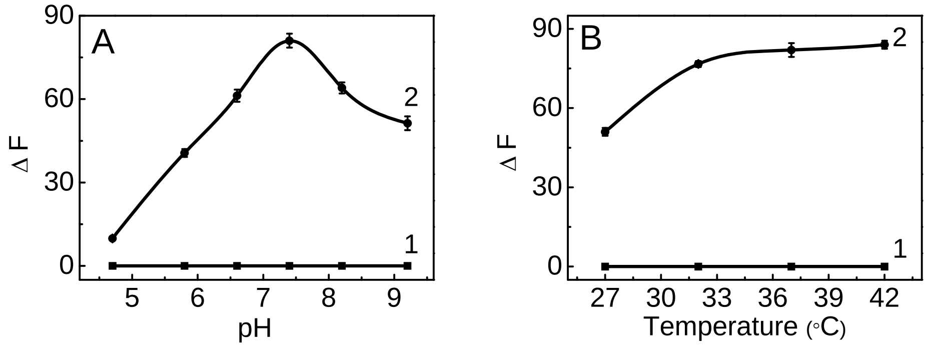

Fig. S5 Effects of (A) pH and (B) reaction temperature on the fluorescence of 5 μM probe **1** (1) without and (2) with PGP-1 (1.5 μg/mL). Conditions: (A) the reaction was performed in 6.7 mM PBS for 30 min at different pH values adjusted with dilute HCl or NaOH; (B) the reaction was performed in 6.7 mM PBS (pH 7.4) for 30 min at different temperatures. $\lambda_{ex/em}$ = 585/625 nm. As can be seen, PGP-1 functions well under the physiological conditions (pH 7.4, 37 °C).

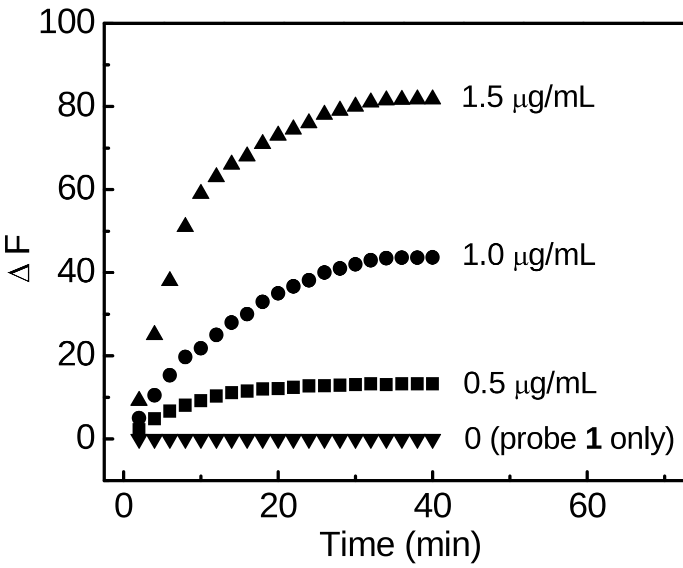

Fig. S6 Effects of reaction time on the fluorescence enhancement of probe **1** (5 μM) in the presence of varied concentrations of PGP-1 (0-1.5 μg/mL). The reaction was performed at 37 °C in 6.7 mM PBS (pH 7.4). $\lambda_{ex/em}$ = 585/625 nm. As seen, a reaction time of 30 min may be chosen.

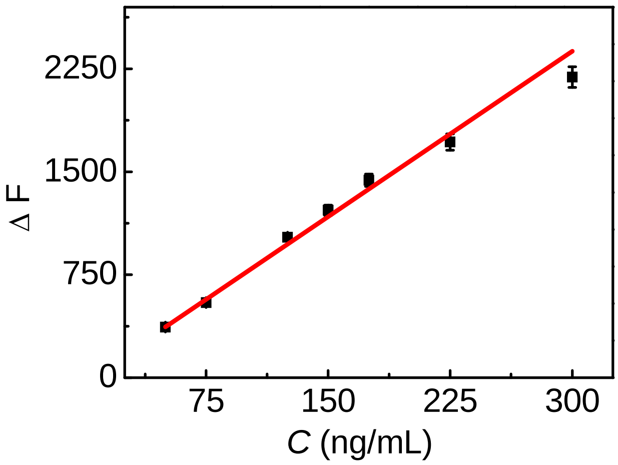

Fig. S7 A plot of fluorescence enhancement of commercial PGP-1 probe (5 μM) versus various concentration of PGP-1. The reaction was performed at 37 °C in 6.7 mM PBS (pH 7.4) for 30 min. $\lambda_{ex/em}$ = 340/440 nm. The detection limit of the commercial PGP-1 probe was determined to be 14.6 ng/mL.

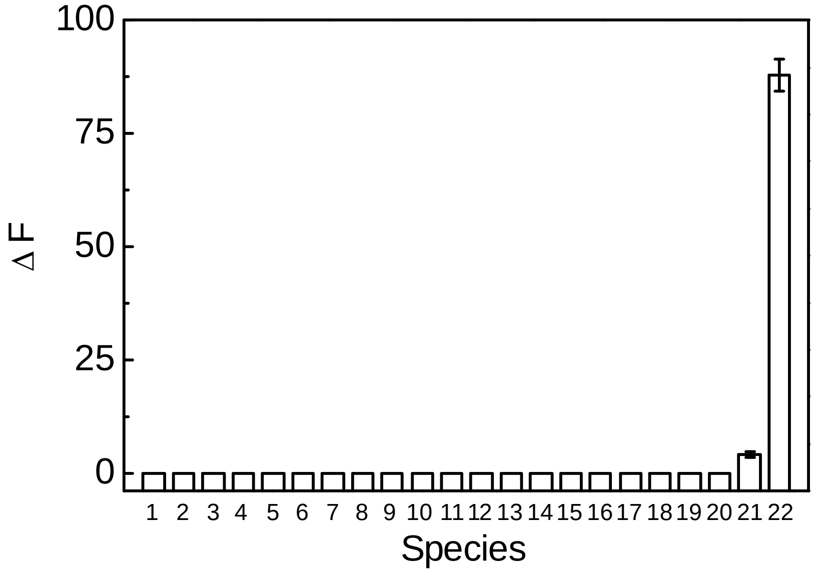

Fig. S8 Fluorescence change of probe **1** (5 μM) in the presence of various species: (1) probe only (control); (2) 150 mM KCl; (3) 2.5 mM $CaCl_2$; (4) 100 μM $ZnCl_2$; (5) 2.5 mM $MgCl_2$; (6) 100 μM $CuSO_4$; (7) 10 mM glucose; (8) 1 mM vitamin C; (9) 1 mM glutamic acid; (10) 1 mM cysteine; (11) 5 mM glutathione; (12) 100 μM ClO^-; (13) 100 μM human serum albumin; (14) 4 ng/mL LAP; (15) 2 μg/mL V8 protease; (16) 2 μg/mL prolidase; (17) 2 μg/mL esterase; (18) 2 μg/mL trypsin; (19) 2 μg/mL thrombin; (20) 2 μg/mL DPPIV; (21) 2 μg/mL PGP-2; (22) 2 μg/mL PGP-1.

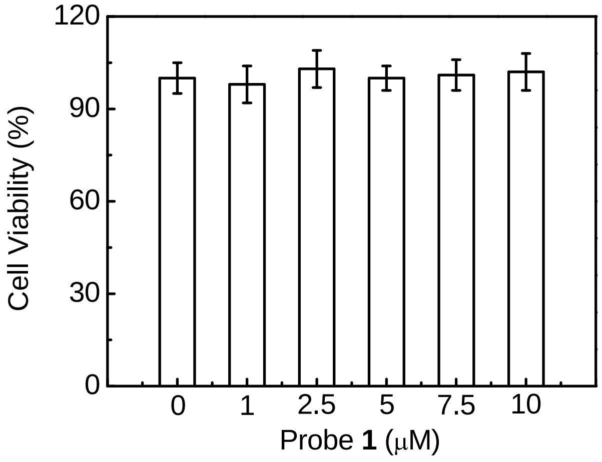

Fig. S9 Effects of probe **1** at varied concentrations on the viability of LO-2 cells.

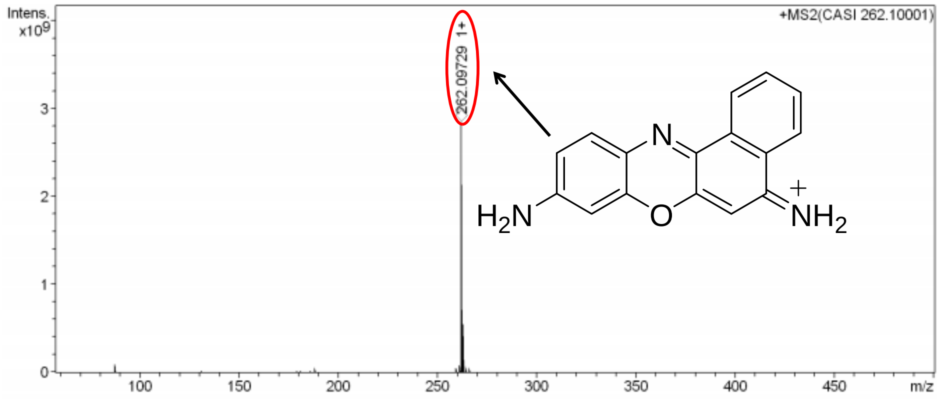

Fig. S10 HR-ESI mass spectrum of the reaction product of **1** with PGP-1.

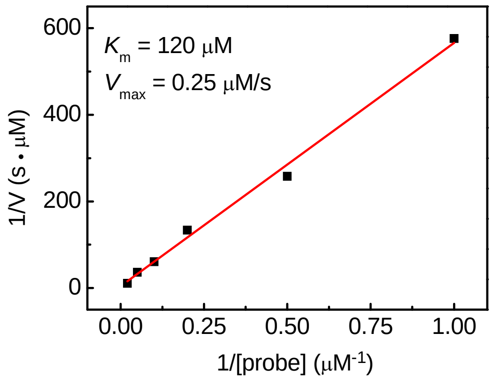

Fig. S11 Lineweaver-Burk plot for the enzyme-catalyzed reaction. The Michaelis-Menten equation was described as: $V = V_{max}[\text{probe}]/(K_m+[\text{probe}])$, where V is the reaction rate, [probe] is the probe concentration, and K_m is the Michaelis constant. Conditions: 900 ng/mL PGP-1, 1-50 μM probe **1**, pH 7.4 PBS, temperature 37 °C. $\lambda_{ex/em}$ = 585/625 nm.

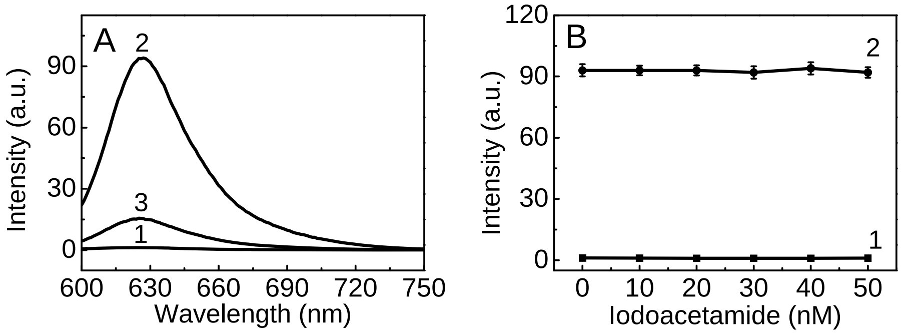

Fig. S12 (A) Fluorescence emission spectra of different reaction systems. (1) Probe **1** only (5 μM); (2) probe **1** (5 μM) + PGP-1 (2 μg/mL); (3) probe **1** (5 μM) + PGP-1 (2 μg/mL) + iodoacetamide (50 nM). λ_{ex} = 585 nm. As is seen, addition of the inhibitor (iodoacetamide)

largely decreases the fluorescence of the reaction system via inhibiting the PGP-1 activity. (B) The effects of iodoacetamide at varied concentrations on the fluorescence intensity of (1) probe **1** (5 μM) and (2) cresyl violet (5 μM). The reactions were performed at 37 °C for 30 min in 6.7 mM PBS (pH 7.4). $\lambda_{ex/em}$ = 585/625 nm. As is seen, the presence of iodoacetamide does not affect the fluorescence of probe **1** and its reaction product.

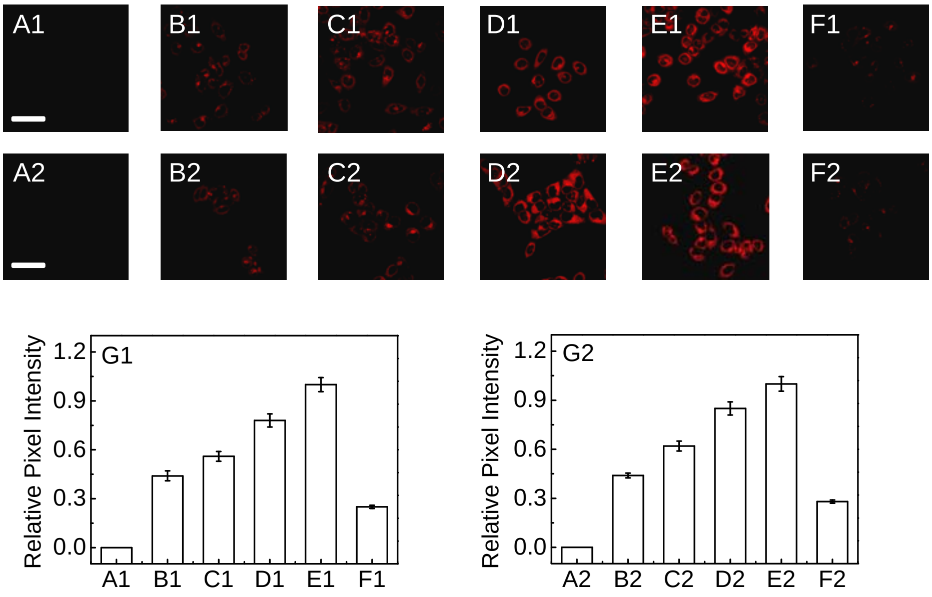

Fig. S13 Fluorescence images of LO-2 cells. (A1, A2) LO-2 cells only. (B1-E1) The LO-2 cells were pretreated with 0.25 % (v/v) FIA at 37 °C for 0, 4, 14 and 24 h, respectively, and then incubated with the probe (5 μM) for 30 min. (F1) LO-2 cells pretreated with 0.25 % FIA for 24 h, then treated with 50 nM iodoacetamide for 1 h, and finally incubated with the probe (5 μM) for 30 min. (B2-E2) LO-2 cells pretreated with 1.0 μg/mL LPS at 37 °C for 0, 4, 12 and 16 h, respectively, and then incubated with the probe (5 μM) for 30 min. (F2) LO-2 cells pretreated with 1.0 μg/mL LPS for 16 h, then treated with 50 nM iodoacetamide for 1 h, and finally incubated with the probe (5 μM) for 30 min. (G1, G2) Relative pixel intensity measurements (n = 3) from the above corresponding images by using software ImageJ (the pixel intensities from images E1 and E2 are defined as 1.0). Scale bar 20 μm. As is seen, increase of incubation time of FIA and LPS leads to the gradual increase of cellular fluorescence intensity via the up-regulation of intracellular PGP-1, and introduction of iodoacetamide largely decreases the intracellular fluorescence by inhibiting the activity of PGP-1. Note that incubation time of 24 h and 16 h for FIA and LPS, respectively, was used in subsequent experiments.

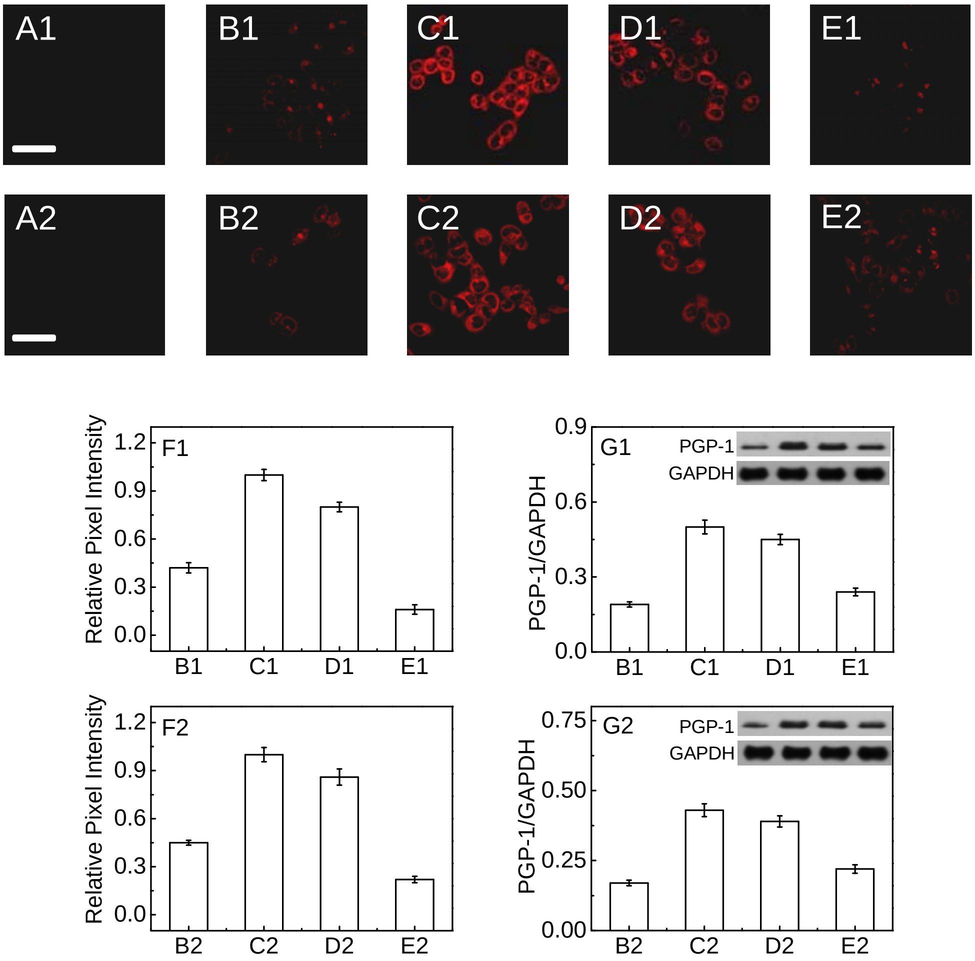

Fig. S14 Fluorescence images of LO-2 cells and their western blot assay. (A1, A2) LO-2 cells only. (B1, C1) The LO-2 cells were pretreated with 0.25 % FIA at 37 °C for 0 and 24 h, respectively, and then incubated with the probe (5 μM) for 30 min. (B2, C2) The LO-2 cells were pretreated with 1.0 μg/mL LPS for 0 and 16 h, respectively, and then incubated with the probe (5 μM) for 30 min. (D1, E1) The LO-2 cells were pretreated with 0.25 % FIA for 24 h, then incubated with iodoacetamide at 5 nM and 50 nM, respectively, for 1 h, and finally incubated with the probe (5 μM) for 30 min. (D2, E2) The LO-2 cells were pretreated with 1.0 μg/mL LPS for 16 h, then incubated with iodoacetamide at 5 nM and 50 nM, respectively, for 1 h, and finally incubated with the probe (5 μM) for 30 min. (F1, F2) Relative pixel intensity measurements (n = 3) from the above corresponding images by using software ImageJ (the pixel intensities from images C1 and C2 are defined as 1.0). (G1, G2) The changes of PGP-1 in the above LO-2 cells determined by western blot (the molecular weight of PGP-1 was determined to be 23 kDa).

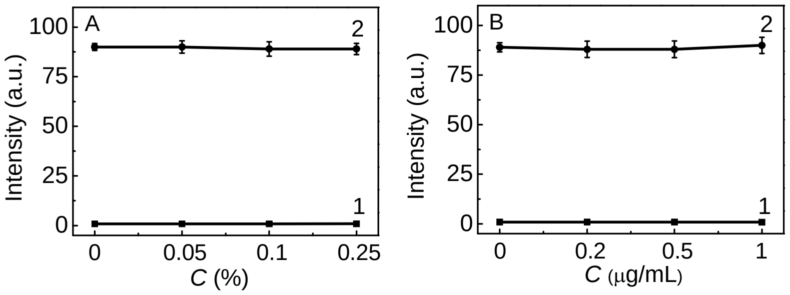

Fig. S15 Effects of (A) FIA and (B) LPS on the fluorescence of (1) the probe (5 μM) and (2) its reaction with PGP-1 (2 μg/mL). All the reactions were performed in 6.7 mM PBS (pH 7.4) at 37 °C for 30 min. $\lambda_{ex/em}$ = 585/625 nm.

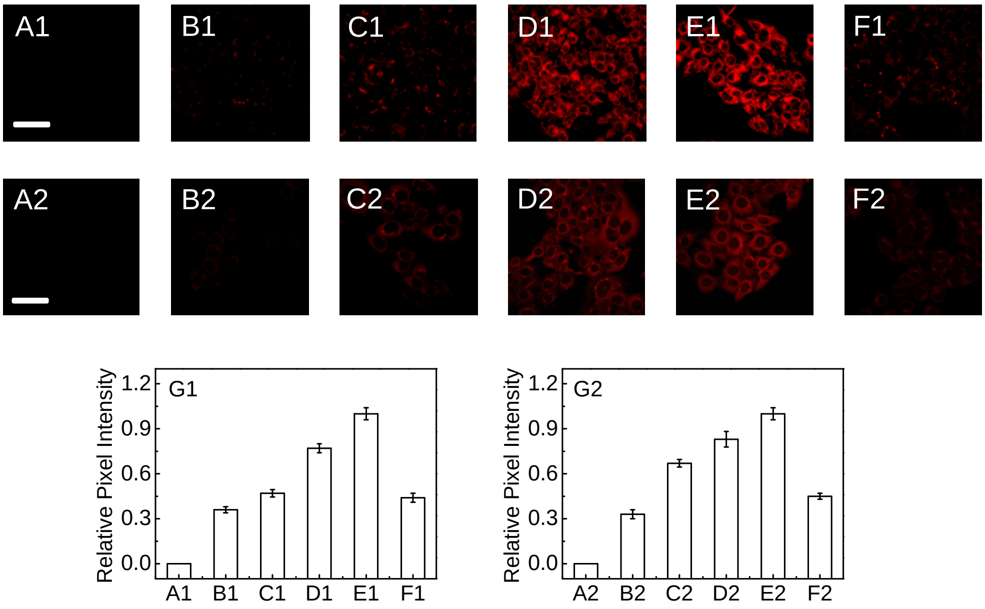

Fig. S16 Fluorescence images of PGP-1 in HepG2 cells. (A1, A2) HepG2 cells only. (B1-E1) The HepG2 cells were pretreated with FIA at varied concentrations (0, 0.05, 0.1, 0.25%, respectively) at 37 °C for 24 h, and then incubated with the probe (5 μM) for 30 min. (F1) The HepG2 cells were pretreated with 0.25% FIA, then with 50 nM iodoacetamide for 1 h, and finally incubated with the probe (5 μM) as above. (B2-E2) The HepG2 cells were pretreated with LPS at varied concentrations (0, 0.2, 0.5, 1.0 μg/mL, respectively) for 16 h, and then incubated with the probe (5 μM) for 30 min. (F2) The HepG2 cells were pretreated with 1.0 μg/mL LPS, then with 50 nM iodoacetamide for 1 h, and finally incubated with the probe (5 μM) as above. (G1, G2) Relative pixel intensities measurements (n = 3) from the above corresponding images by using software ImageJ (the pixel intensities from images E1 and E2 are defined as 1.0). Scale bar, 20 μm.
